# Research on the Structural Design and Mechanical Properties of T800 Carbon Fiber Composite Materials in Flapping Wings

**DOI:** 10.3390/ma18153474

**Published:** 2025-07-24

**Authors:** Ruojun Wang, Zengyan Jiang, Yuan Zhang, Luyao Fan, Weilong Yin

**Affiliations:** 1 Center for Composite Materials and Structures, Harbin Institute of Technology, Harbin 150080, China; 23s118135@stu.hit.edu.cn (R.W.); 24s118100@stu.hit.edu.cn (L.F.); 2 Aerodynamics Research Institute, AVIC (Aviation Industry Corporation of China), Harbin 150080, China; songbinghao00001@126.com

**Keywords:** micro flapping-wing aircraft, T800 carbon fiber composite material, finite element analysis, aerodynamic characteristics, 3D printing

## Abstract

Due to its superior maneuverability and concealment, the micro flapping-wing aircraft has great application prospects in both military and civilian fields. However, the development and optimization of lightweight materials have always been the key factors limiting performance enhancement. This paper designs the flapping mechanism of a single-degree-of-freedom miniature flapping wing aircraft. In this study, T800 carbon fiber composite material was used as the frame material. Three typical wing membrane materials, namely polyethylene terephthalate (PET), polyimide (PI), and non-woven kite fabric, were selected for comparative analysis. Three flapping wing configurations with different stiffness were proposed. These wings adopted carbon fiber composite material frames. The wing membrane material is bonded to the frame through a coating. Inspired by bionics, a flapping wing that mimics the membrane vein structure of insect wings is designed. By changing the type of membrane material and the distribution of carbon fiber composite materials on the wing, the stiffness of the flapping wing can be controlled, thereby affecting the mechanical properties of the flapping wing aircraft. The modal analysis of the flapping-wing structure was conducted using the finite element analysis method, and the experimental prototype was fabricated by using 3D printing technology. To evaluate the influence of different wing membrane materials on lift performance, a high-precision force measurement experimental platform was built, systematic tests were carried out, and the lift characteristics under different flapping frequencies were analyzed. Through computational modeling and experiments, it has been proven that under the same flapping wing frequency, the T800 carbon fiber composite material frame can significantly improve the stiffness and durability of the flapping wing. In addition, the selection of wing membrane materials has a significant impact on lift performance. Among the test materials, the PET wing film demonstrated excellent stability and lift performance under high-frequency conditions. This research provides crucial experimental evidence for the optimal selection of wing membrane materials for micro flapping-wing aircraft, verifies the application potential of T800 carbon fiber composite materials in micro flapping-wing aircraft, and opens up new avenues for the application of advanced composite materials in high-performance micro flapping-wing aircraft.

## 1. Introduction

The design inspiration for flapping-wing aircraft stems from the biomimetic research on the flight mechanisms of birds and insects in nature [1]. Compared to traditional fixed-wing and rotor-wing aircraft, flapping-wing aircraft can precisely replicate the periodic flapping motion and three-dimensional twisting motion of biological wings, achieving efficient aerodynamic performance in low Reynolds number fluid environments [2]. The biomimetic structure design endows it with excellent adaptability to complex environments, enabling stable flight in confined spaces and possessing high maneuverability, low noise, and other advantages [3]. These characteristics mean that flapping-wing aircraft have significant application value in the field of micro unmanned aircraft [4,5].

Lightweight technology, as a key approach to enhancing the comprehensive performance of aircraft, optimizing energy utilization efficiency, and increasing effective carrying capacity, has always been the core of research in the aerospace field [6]. Therefore, weight is one of the important indicators for measuring the advancedness of aircraft design. Under the premise of meeting flight tasks, reducing the weight of aircraft is the eternal pursuit of designers. If lightweight design is applied to flapping-wing aircraft, it means a lighter fuselage, less fuel consumption, greater flexibility and convenience, and thus greater market prospects. Therefore, we hope to design flapping-wing aircraft with lightweight structures under the premise of meeting certain strength, stiffness, and lifespan requirements. There are mainly two ways of lightweighting: one is structural optimization [7,8], and the other is the use of stronger materials, especially advanced composite materials [9,10,11].

Structure is the skeleton of aerospace equipment, and for a long time, it has constituted the main object of system lightweighting. Lightweighting of material structure requires the full and reasonable application of high-performance lightweight materials (such as lightweight alloys, composite materials, foam/foam core/particle lattice materials, etc.) [12], new structural optimization design methods (topology optimization, overall optimization, etc.) [13,14] and new process technologies (additive manufacturing, composite manufacturing, etc.), achieving the optimization and lightweighting of multiple carrying performances through the rational layout and parameter optimization of materials in the structural space.

With the rapid development of biomimetic aircraft technology, flapping-wing aircraft, due to their efficient aerodynamic characteristics and biomimetic maneuverability, have become a research hotspot. Traditional metal materials are more commonly used in large aircraft, but in the field of flapping-wing aircraft, they face challenges such as heavy weight, insufficient fatigue performance, etc., in achieving high lift, low energy consumption and long lifespan. Composites, with their lightweight, high specific strength, excellent fatigue resistance and designability, provide new ideas for flapping-wing aircraft structure design and performance improvement, and have promoted significant progress in the research of micro flapping-wing aircraft. The application of composite materials in modern flapping-wing aircraft mainly lies in weight reduction, aerodynamic optimization and functional integration [15,16]. In terms of weight reduction, carbon fiber reinforced composite materials have extremely high strength and low density and are widely used [17,18]. In terms of aerodynamic optimization, resin-based composite materials, through integrated molding processes, have high precision characteristics and have broad application prospects in micro flapping-wing aircraft. International research on composite materials in flapping-wing aircraft has formed a relatively standardized integration model, mainly concentrated in research institutions in the United States, Japan and Europe. The US DARPA proposed the concept of micro aircraft in 1992, promoting the application of composite materials in micro flapping-wing aircraft [19]. The Robobees series from Harvard University employs the Smart Composite Microstructure (SCM) technology, embedding piezoelectric materials into the composite wing structure, achieving an ultra-miniature design with a 3 cm wingspan and 80 mg weight. The latest generation of Robobee X-Wing, through the optimization of the four-wing structure and lightweight design of the composite material, weighs 259 mg and has a thrust efficiency close to that of insects of the same volume [20,21]. The Defly Nimble flapping robot from Delft University of Technology uses transparent polyester film composite wing structures, achieving agile flight movements similar to those of fruit flies, including sharp turns and back flips at a speed of 25 km/h. This design fully demonstrates the advantages of composite materials in achieving complex kinematics [22].

In the field of micro-bionic flapping-wing aircraft, polymer materials, due to their low modulus characteristics, can meet the requirements for the high-frequency reciprocating motion of the flapping wings. Polymer materials also have excellent specific strength and fatigue resistance, which can ensure the structural stability of the mechanism under long-term cyclic loading. Therefore, polymer materials can be used as the wing surface framework to achieve lightweight configuration design, significantly reducing energy loss during the flight process. Their excellent molding processability is applied in 3D printing, injection molding and other processes, enabling the high-precision fabrication of complex bionic structures [23,24]. Advanced composite materials formed by combining high-performance reinforcing elements such as carbon fibers and glass fibers with polymer matrices can effectively meet the dual performance requirements of flapping-wing structures for flexible deformation ability and structural stiffness. The stiffness of the wing shape (including rigid wings and flexible wings) of flapping-wing aircraft has a decisive impact on its aerodynamic performance. In recent years, scholars at home and abroad have conducted in-depth research on the energy efficiency and biomimetic optimization of these two types of wing shapes, with the aim of breaking through the performance limitations of traditional aircraft [25,26,27]. Through structural optimization design guided by bionics principles, such composite materials can further improve the aerodynamic efficiency of the flapping system and significantly enhance key performance parameters such as flapping frequency. Studies have shown that this material structure integrated design method not only solves the aerodynamic efficiency bottleneck problem of traditional aircraft in low Reynolds number conditions but also provides a new technical path for the performance improvement of micro aircraft. Especially in scenarios that require both lightweighting and structural strength, composite flapping-wing aircraft demonstrate irreplaceable technical advantages [28,29,30,31].

## 2. Materials and Methods

### Single-Degree-of-Freedom Fluttering Mechanism

**Figure 1:**
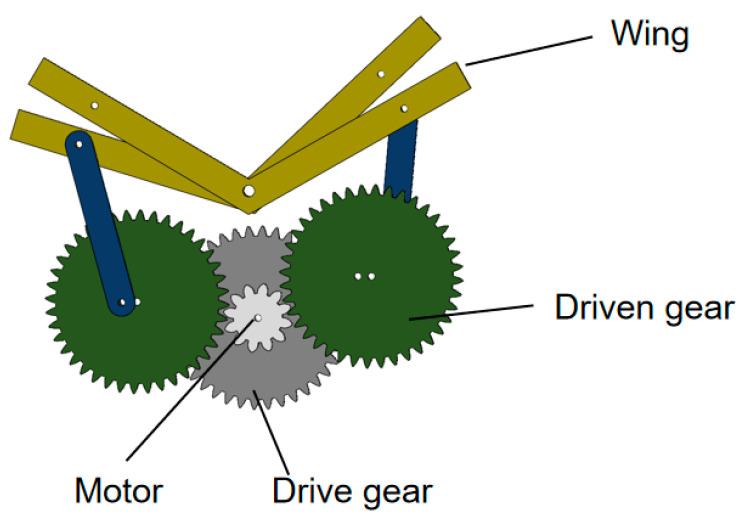
Schematic diagram of single-degree-of-freedom flapping mechanism.

To investigate the influence of different wing membrane materials on the aerodynamic characteristics of bionic aircraft, this study designed a single-degree-of-freedom flapping mechanism, as illustrated in Figure 1. Based on this mechanism, experimental research was conducted. This mechanism achieves flapping motion through a single driving source, which significantly reduces mechanical complexity and enhances the system’s power-to-weight ratio, thereby fulfilling the lightweight design requirements for bionic aircraft. The simplified structure effectively avoids coordination control and motion coupling issues inherent in multi-degree-of-freedom systems, enabling rapid construction and debugging while minimizing processing costs, assembly errors, and potential failure points. Consequently, the stability and repeatability of the experimental system are improved. Moreover, the single-degree-of-freedom design facilitates precise control over key motion parameters such as flapping frequency and amplitude.

In this study, the simplified modeling method was adopted to conduct static simulation analysis on the gear transmission mechanism. Based on the local effect assumption of saint venant principle [32], the influence of secondary structural components far from the load action area on the gear contact stress distribution can be ignored. Therefore, when conducting strength checks on a single-degree-of-freedom fluttering mechanism, the connecting rod structure can be omitted, and the analysis object can be simplified to a transmission system composed of three meshing gears. This modeling simplification strategy can effectively reduce the model complexity. On the premise of ensuring the calculation accuracy of key mechanical parameters such as tooth surface contact stress and tooth root bending stress [33], it realizes the reasonable allocation of computing resources to the mesh refinement of the gear meshing area.

**Figure 2:**
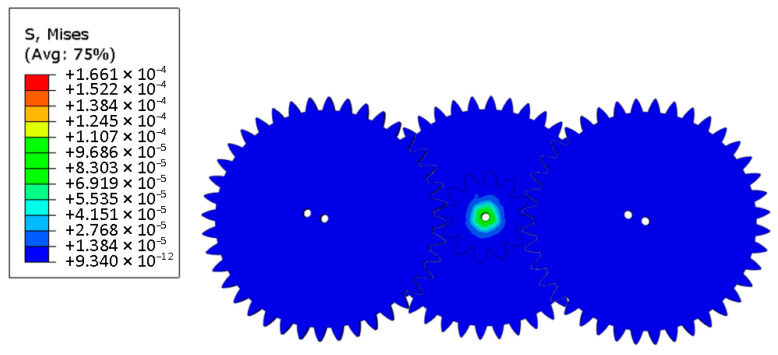
Cloud diagram of stress verification for gear mechanism.

In order to evaluate whether the use of nylon material meets the requirements of gear strength, a finite element analysis model was established based on the designed single-degree-of-freedom flapping mechanism. As shown in the figure, in this study, the numerical model of the gear meshing part of the flapping mechanism was constructed by using the Abaqus/Standard (2020) commercial finite element analysis software. The geometric nonlinear effect (based on the finite deformation theory) was considered in the analysis process. The model parameters are set as follows: The module of the driving gear connected to the drive motor is 0.3 and the number of teeth is 9. The modules of the two driven gears are both 0.3 and the number of teeth is both 40. The model was meshed by using the C3D8R element type, and the strength of the mechanism was checked through stress analysis. The simulation analysis results show that, as shown in Figure 2, the maximum stress value of the flinging mechanism is 166.1 Pa, and its position is located in the connection area between the driving gear and the motor. This stress value is significantly lower than the yield strength limit of the nylon material. From this, it can be determined that the selected nylon material meets the design requirements in terms of strength performance and has reliable mechanical load-bearing capacity.

## 3. Preparation of Flapping Wing Prototype

### 3.1. Flapping Wing Shape Design

**Figure 3:**
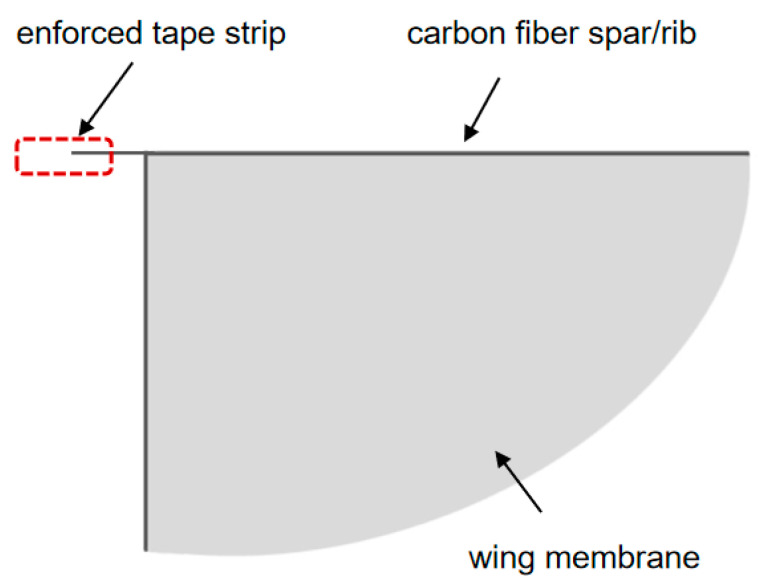
Semi-enveloped wing configuration.

Based on the above finite element analysis results, an improved design was carried out based on the wing membrane and wing vein. For the lightweight design of the wing, the wing shape was first designed according to the aerodynamic performance. The rigid front is composed of carbon fiber rods, and the flexible wing is composed of polyester film, as shown in Figure 3. The trailing edge of the wing is fixed on the fuselage to ensure that the flexible material of the wing surface can undergo significant deformation during the flapping process of the wing. To a certain extent, the torsional movement of the wings is achieved, enabling the prototype to obtain better aerodynamic performance. Based on the wing design of “wing membrane plus wing vein”, carbon fiber rods are used to wrap the entire wing membrane around it to enhance rigidity. The designed mechanism is connected to the flapping mechanism. With this scheme, the wing rigidity increases.

**Figure 4:**
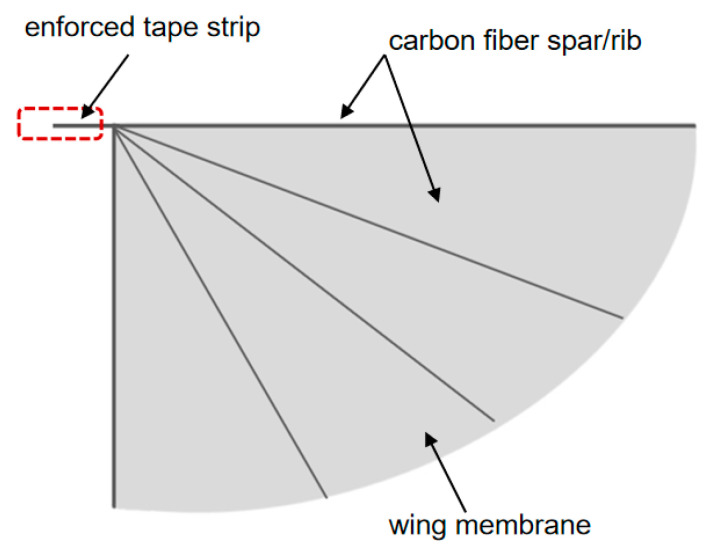
Schematic diagram of the wing membrane-wing vein structure.

**Figure 5:**
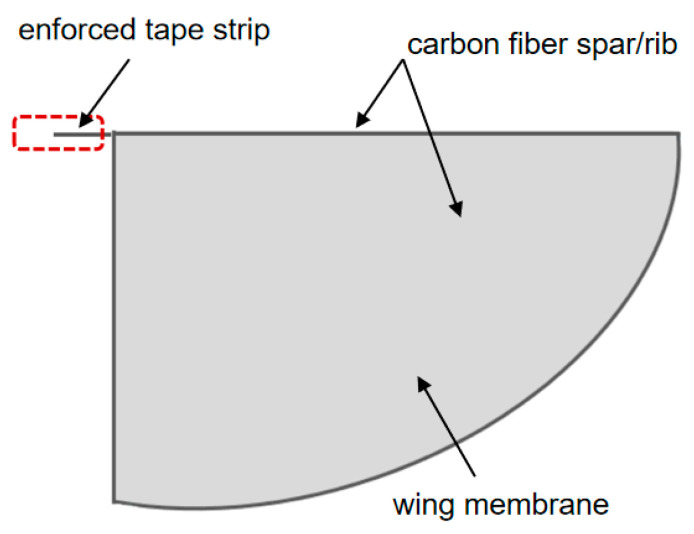
Fully enclosed flapping-wing structure.

In order to imitate the morphological and functional characteristics of natural biological wings more accurately, this paper proposes a bionic airfoil design scheme based on the composite structure of “wing membrane-wing vein”, as shown in Figure 4. In terms of the selection of wing surface materials, polyester film with a thickness of 0.0125 mm was used as the main load-bearing surface. The ultra-thin polyester film not only provides a certain strength but also minimizes the weight of the wing itself to the greatest extent. To enhance the aerodynamic performance of the airfoil mechanism, carbon fiber reinforced composite materials with diameters of 1 mm are used to support the wing surfaces at the leading edge and the wing root of the wing, as shown in Figure 5. The passive pitch motion during the flapping process is guaranteed through the sleeve type non-fixed connection method. This design effectively simulates the adaptive deformation characteristics of natural wings during the flapping process.

**Figure 6:**
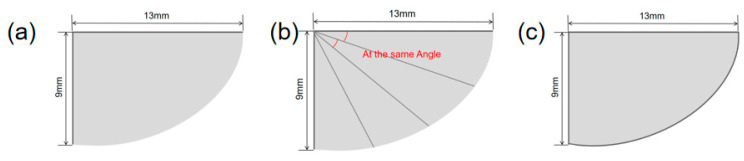
(**a**) Wing configuration 1 (**b**) wing configuration 2 (**c**) wing configuration 3.

This study conducted a modal analysis of the wing structure. During the modeling process, the geometric shape of the wing was simplified to an elliptical wing surface structure. Three different wing structures were designed, as shown in Figure 6. The wing vein structure was simulated using T800 carbon fiber rods, and its material parameters were as follows: the density was 1.8 g/cm^3^, the elastic modulus was 300 GPa, and the Poisson’s ratio was 0.3. The finned membrane structure was simulated using PET film material, and its material parameters were density 1.323 g/cm^3^, elastic modulus 28 GPa, and Poisson’s ratio 0.3. In the process of finite Element modeling, Shell elements are selected for discretization processing, and no additional constraint conditions need to be applied.

**Figure 7:**
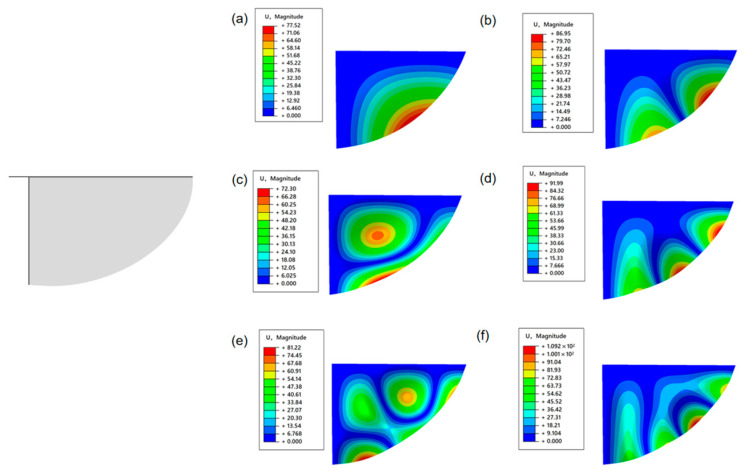
Semi-enveloped wing configuration—the first six vibration modes of the elliptical wings (**a**) C1-first-order mode (vibration in the middle section of the wing). (**b**) C1-The second-order mode (the vibration intensity in the wings has increased). (**c**) C1-The third-order mode (the vibration area spreads towards the middle of the wing). (**d**) C1-The fourth-order mode (coupling vibration occurs). (**e**) C1-The fifth-order mode (the coupling vibration intensity has increased). (**f**) C1-The sixth-order mode (coupled vibrations are obvious).

As can be seen from Figure 7, the first six natural frequencies of the fixed two constrained elliptical wings on both sides are 0.11386 Hz, 0.26436 Hz, 0.40122 Hz, 0.48529 Hz, 0.69106 Hz, and 0.77702 Hz, respectively. The simulation results of the first six vibration modes of the elliptical wing are shown in the following figure. It can be seen that the vibrations all occur at the posterior edge of the elliptical wings. Since the wing veins at the posterior edge have no wing veins and the bending stiffness is very low, this area is prone to inducing vibrations.

**Figure 8:**
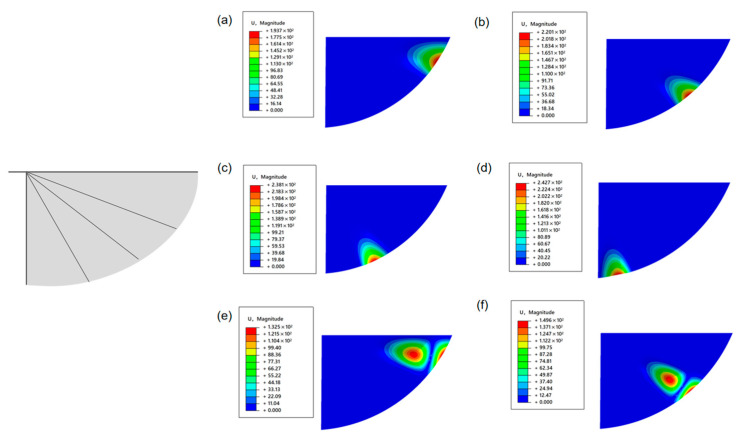
Schematic diagram of the wing membrane-wing vein structure—the first six vibration modes of the elliptical wings. (**a**) C2-first-order mode (low-order vibration in the aft edge area of the wing tip). (**b**) C2-The second-order mode (vibration at the middle part of the wing’s trailing edge). (**c**) C2-The third-order mode (the vibration spreads from the rear edge root of the wing). (**d**) C2-The fourth-order mode (vibration in the wing root area). (**e**) C2-The fifth-order mode (Wingtip coupling vibration). (**f**) C2-The sixth-order mode (coupled vibration of the wing trailing edge).

Imitating the wing membrane-wing vein structure of insect wings, T800 carbon fiber rods are introduced into the model as wing vein materials to enhance rigidity. As can be seen from Figure 8, the natural frequencies of the first six orders of the optimized structure have significantly increased, which are 1.1551 Hz, 1.3692 Hz, 1.5081 Hz, 1.7587 Hz, 2.1190 Hz and 2.2870 Hz, respectively. The mode analysis shows that, although the vibration still mainly occurs at the trailing edge of the wing, the vibration area is significantly reduced, indicating that the introduction of the wing veins effectively enhances the local stiffness and suppresses the overall vibration response of the structure.

**Figure 9:**
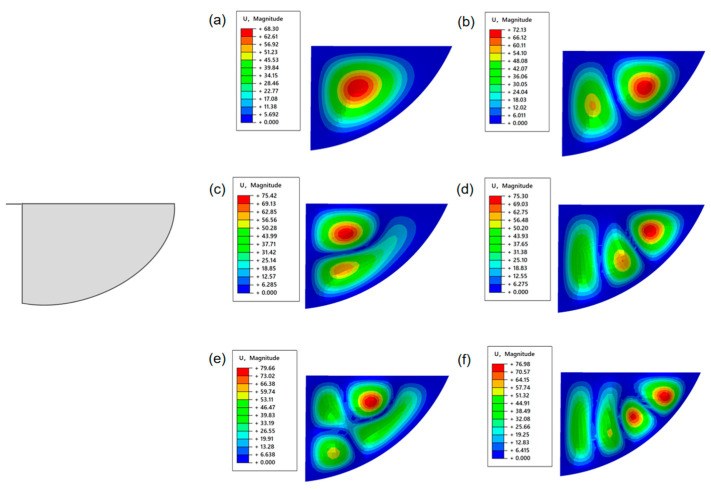
Fully enclosed flapping-wing structure—the first six vibration modes of the elliptical wings. (**a**) C3-first-order mode (vibration in the middle part of the wing). (**b**) C3-The second-order mode (coupled vibration occurs in the middle part of the wing). (**c**) C3-The third-order mode (the vibration intensity in the middle part of the wing has increased). (**d**) C3-The fourth-order mode (the vibration in the middle part of the wing is obvious). (**e**) C3-The fifth-order mode (coupled vibration in the middle section of the wing) (**f**) C3-The sixth-order mode (high-order coupled vibration in the middle section of the wing).

As can be seen from Figure 9, the entire area around the elliptical wing was fixed, and the first six natural frequencies were increased to 0.40086 Hz, 0.67604 Hz, 0.89124 Hz, 1.0339 Hz, 1.2965 Hz, and 1.4711 Hz. It can be seen that the vibration concentration area shifted to the middle of the wing. The reason is that no wing vein support was set in this area. The relatively low structural stiffness makes it a sensitive area of the vibration-dominated mode.

This study shows that the introduction of wing vein materials can significantly enhance the overall stiffness of the structure, thereby significantly increasing the natural frequency. In flapping-wing structures, the low-stiffness areas (such as the trailing edge or middle part without wing vein support) are more prone to form vibration concentration areas due to their weaker bending resistance. By optimizing the spatial arrangement of high-stiffness materials (such as wing veins), effective regulation of vibration modes can be achieved, thereby reducing the vibration amplitude and its influence range.

### 3.2. Selection of Flapping Wing Materials

The wing materials of flexible flapping-wing aircraft typically include two types: wing ribs for enhancing stiffness and lightweight wing membranes. The selection of these materials should aim to achieve lightweight structure and functional synergy while meeting mechanical and aerodynamic performance requirements. The wing is a movable part. The quality of it will directly affect the magnitude of its moment of inertia. For moving parts, we should reduce their weight to decrease the inertia of movement. Density is the first factor we need to consider. Apart from the drive power supply, circuit board, coil and other components, the fuselage frame is the component with the largest number of parts and also the one that accounts for the largest proportion of weight. Wing ribs mainly serve as structural supports, and must have high specific strength and specific stiffness to effectively reduce weight while maintaining rigidity, thereby reducing the rotational inertia of the wings during flight and improving maneuverability. Therefore, in order to ensure that the mass of the entire flapping wing device is as small as possible, the density of the selected frame material should also be as low as possible. In order to increase the rigidity of the wing, the wing vein structure needs to be introduced, which should have a certain strength and be able to withstand deformation. Selecting materials with a lower density to manufacture the wing vein components can achieve a smaller wing mass. T800 carbon fiber composite material features low density (1.6 g/cm^3^), extremely high tensile strength and rigidity, which can significantly reduce the weight of the structure, while enhancing the anti-deformation capacity and durability of the wings and structural frames, thereby improving the structural stability of the aircraft [34,35,36]. In addition, T800 material has good designability and its performance can be further optimized by adjusting the arrangement of fibers. With its outstanding mechanical properties and lightweight characteristics, T800 carbon fiber composite material is highly suitable for application in the design of micro aircraft (MAVs) that have high requirements for performance and weight. Therefore, in this paper, T800 carbon fiber composite material is selected as the wing frame material to meet the requirements of structural strength and lightweight. Considering the above points comprehensively, T800 carbon fiber with a diameter of 1mm is selected as the wing frame structure.

In the structural design of this project, the wing membrane is adhered to the wing ribs and directly participates in the transmission of aerodynamic loads. The material of the wing membrane must have good flexibility and airtightness to withstand periodic large deformations of flapping motion and maintain the stability of lift output. To ensure the long-term reliable operation of the overall structure under complex conditions, the selection of the wing membrane material should meet the following requirements: first, low density to reduce wing surface mass and optimize dynamic response; second, excellent adhesion to enhance the connection stability between the membrane material and the framework; third, good flexibility to adapt to elastic deformation during flight; fourth, shape retention ability. To more closely resemble the shape of an insect’s wing, the wing surface should be as close as possible to the biomimetic shape. Therefore, after production, the wing membrane material should not be prone to deformation to maintain its expected aerodynamic shape. These performance requirements collectively form the design basis for the selection of flexible wing materials.

**Figure 10:**
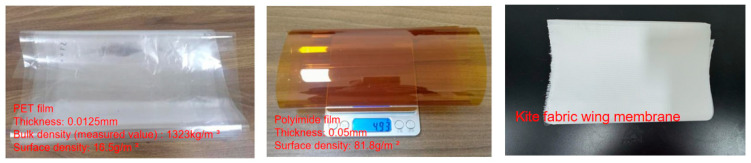
Three types of wing membrane materials.

When designing flap-wing aircraft, PET film materials, polyimide film materials and non-woven kite cloth are mainly selected for experimental comparison, with the aim of choosing wing film materials with good performance and light weight. The surface energy of PET film is relatively low, and it is hydrophobic and chemically inert [37]. The weak Lewis acid-base properties of the PET film surface imply that the interfacial interaction force between it and other materials (such as adhesives, composite layers) is relatively small. Polyimide films have a relatively high surface energy, which is conducive to the adhesion between materials and the bonding of interfaces [38], when micro flapping wings are combined with adhesives or composite materials, the Lewis acid-base properties of the polyimide film surface are conducive to the formation of a stable interface layer. Kite cloth film, as a coating or composite material, has a relatively rough surface [39]. These differences have a significant impact on the mechanical properties and interface behavior of the wing membrane. Therefore, these three materials were selected for the design of the wing membrane to explore the influence of materials with different properties on the mechanical properties of the flapping mechanism. The three types of wing membrane materials are shown in Figure 10, and their material parameters are provided in Table 1.

**Figure 11:**
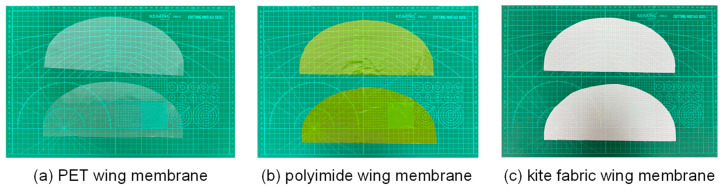
Three kinds of wing membrane cutting and preparation.

This study adopted elliptical wings as the experimental objects. Although automated processing technologies such as laser cutting can achieve high-precision and high-efficiency cutting of wing films, due to the high equipment cost, and the relatively simple processing technology and low manufacturing cost of the flapping wing structure itself, automated processing methods are usually not adopted to handle wing film materials in actual production. Therefore, in this study, the manual cutting method was chosen and a unified template was used to prepare elliptical wing membrane samples. Three kinds of wing membrane materials were selected for the experiment: 0.0125 mm thick PET film, 0.05 mm thick polyimide film and non-woven kite fabric. The samples of three bionic wing membrane materials after cutting and processing are shown in the Figure 11.

**Figure 12:**
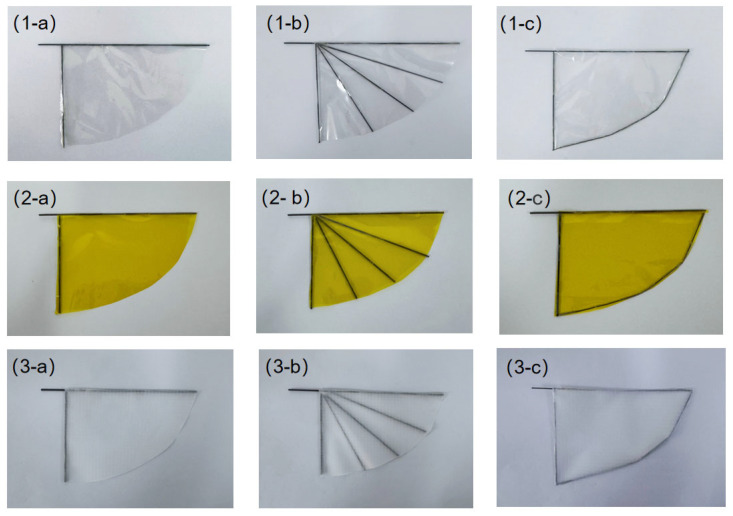
Preparation of three different configurations of wing membranes. (**1-a**) the assembled wing membrane-c1-PET (**1-b**) the assembled wing membrane -c2-PET (**1-c**) the assembled wing membrane- c3-PET (**2-a**) the assembled wing membrane- c1-PI (**2-b**) the assembled wing membrane- c2-PI (**2-c**) the assembled wing membrane -c3-PI (**3-a**) the assembled wing membrane -c1-Kite (**3-b**) the assembled wing membrane- c2-Kite (**3-c**) the assembled wing membrane- c3-Kite.

Three types of wing membrane materials were cut and assembled, respectively, according to the designed configuration. The assembled wing membrane configuration is shown in the Figure 12.

### 3.3. Pulsating Mechanism Printing

**Figure 13:**
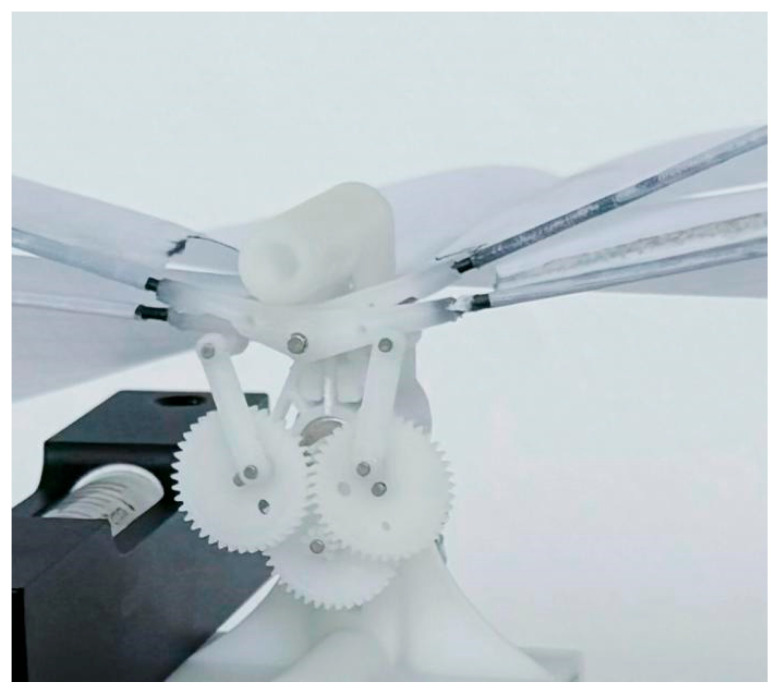
Diagram of the experimental prototype.

The single-degree-of-freedom flutter mechanism designed in this paper is printed using DLP light processing technology. The 3D printer used is the Rayshape P400 (Raise3D, Shanghai, China), which has a high projection resolution of 3840 × 2160 pixels and a printing speed of 15 mm per hour. Resin material is selected for printing. The printed single-degree-of-freedom components are assembled. After assembly, the pre-cut wing membrane material is adhered to the surface of the connection rod of the flutter mechanism with glue. The following Figure 13 shows the prototype of the flutter mechanism used for experimental testing.

## 4. Experiment

### 4.1. Construction of the Force Measurement Experiment Platform

**Figure 14:**
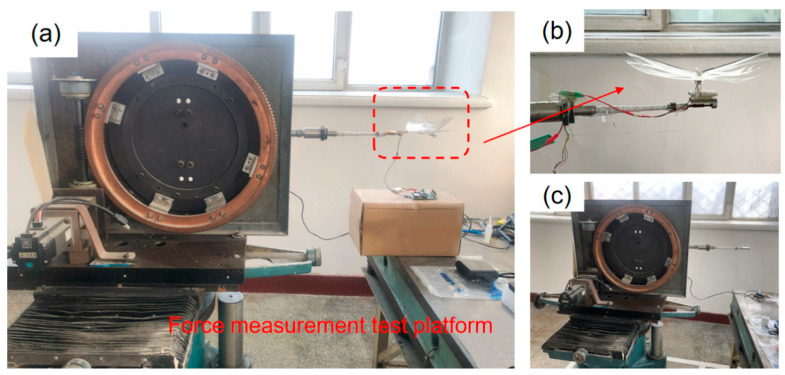
Single-degree-of-freedom force measurement experimental platform (**a**) Schematic diagram of the force measurement experiment process (**b**) Installation diagram of the oscillation mechanism (**c**) Force measurement experimental platform v.

After setting up the experimental platform, data collection was carried out on the force measurement platform of the single-degree-of-freedom flinging mechanism that had already been established. The experimental situation is as shown in the Figure 14. The voltage value range is 0–4 V. According to the control throttle, it is divided into four gears to control the voltage change.

**Figure 15:**
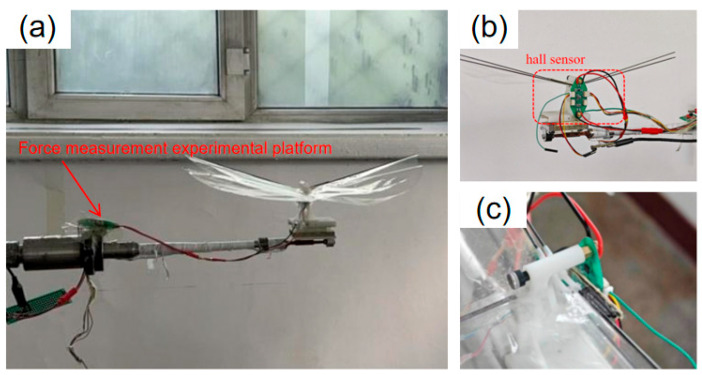
Single-degree-of-freedom Hall sensor experiment. (**a**) The fluttering mechanism installed on the force measurement experimental platform. (**b**) Installation diagram of Hall sensor. (**c**) Local schematic diagram of Hall sensor position.

To achieve precise measurement of the flapping frequency of the single-degree-of-freedom flapping mechanism, a Hall sensor assembly is installed on the mechanism. This sensor is capable of sensing the periodic displacement changes during the movement of the flapping wing, thereby achieving real-time monitoring of the flapping period and calculating the corresponding flapping frequency accordingly. This non-contact measurement method can effectively enhance the stability and reliability of experimental data. The fully built experimental test platform is shown in the Figure 15.

**Figure 16:**
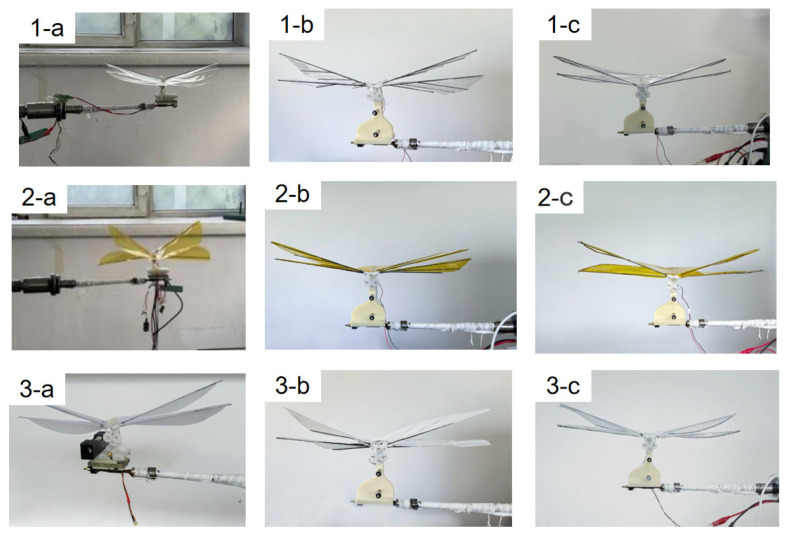
Comparative experiments of three types of wing membrane materials.(**1-a**) c1-PET experimental schematic diagram (**1-b**) c2-PET experimental schematic diagram (**1-c**) c3-PET experimental schematic diagram (**2-a**) c1-PI experimental schematic diagram (**2-b**) c2-PI experimental schematic diagram (**2-c**) c3-PI experimental schematic diagram (**3-a**) c1-Kite experimental schematic diagram (**3-b**) c2-Kite experimental schematic diagram (**3-c**) c3-Kite experimental schematic diagram.

In order to study the influence of different wing membrane materials and different stiffness configurations on the mechanical properties of the single-degree-of-freedom flinging mechanism, under the premise of ensuring the mechanism structure and driving power supply remain unchanged, three different types of wing membrane materials were selected for replacement, and comparative experiments were carried out, respectively. Through comparative experiments, the influence of wing membrane materials on performance indicators such as lift and frequency was evaluated. The process of changing the wing membrane material and the experiment under different configurations is shown in the Figure 16.

### 4.2. Experimental Data Analysis

**Figure 17:**
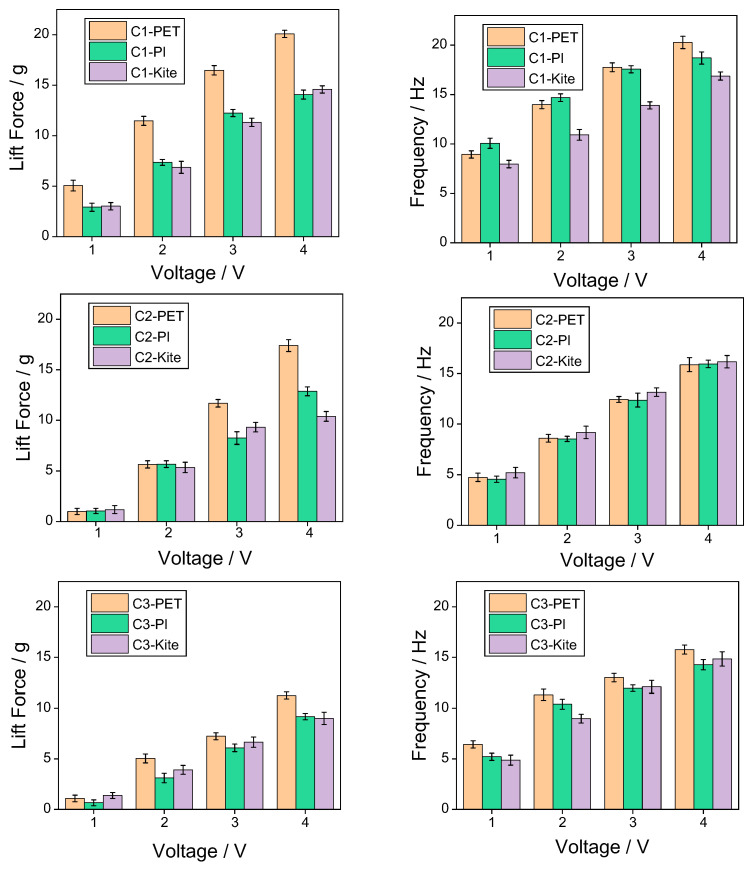
The effects of three different materials on lift and frequency.

**Figure 18:**
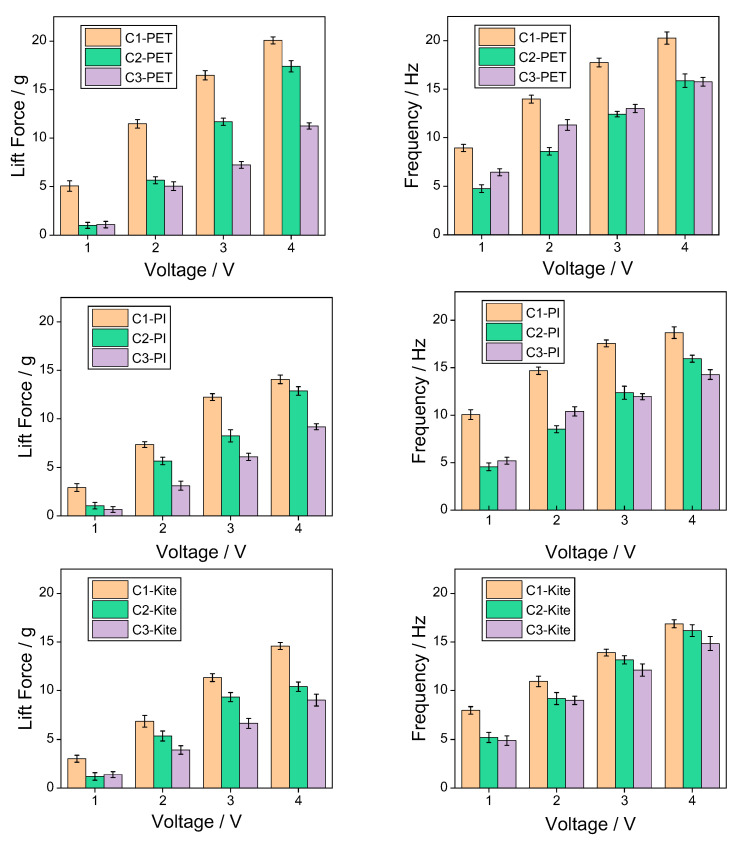
The effects of three different configurations on lift and frequency.

To explore the influence of wing membrane materials and airfoil configurations on the mechanical properties of the flapping mechanism, force measurement and frequency measurement experiments were conducted on three combinations of wing membrane materials and three configurations, respectively. To enhance the reliability and representativeness of the experimental results, each group of experiments was independently measured three times, and the average value of the obtained data was calculated to reduce the influence of accidental errors. The experimental data are shown in the following table. By conducting post-processing on the experimental data and drawing bar graphs, it is convenient to compare the influence of different factors on mechanical properties more intuitively. As shown in Figure 17, it presents the variation laws of lift and flutter frequencies corresponding to different wing membrane materials under the same configuration conditions; Figure 18 shows the variation laws of lift and frequency corresponding to different flapping wing configurations under the same wing membrane material conditions.

From the data in the above Table 2 and Table 3, it can be seen that the highest flapping frequency of 20.27 Hz and the flapping lift of 20.07 g both originated from configuration 1 of the PET film material. It can be seen from Figure 17 that under the same configuration, the lift of the pet film is significantly higher than that of the polyimide film and the kite cloth film. From Figure 18, it can be seen that under low-voltage drive, the lighter structure can generate a relatively large lift force with a smaller voltage input. Although the designs of configuration 2 and configuration 3 offer strong structural stiffness, the additional weight offsets part of the lift effect, resulting in their lift being much lower than that of configuration 1.

In this study, three distinct wing configurations, each paired with three different membrane materials, were systematically designed and evaluated. Force measurement experiments were conducted on the established force measurement platform to evaluate the performance indicators of lift and frequency. Among these designs, the C1-PET configuration, which exhibited the best lift performance, was selected for a comparative analysis with two well-established bio-inspired micro air vehicles: Harvard’s RoboBee and DelFly Nimble from Delft University of Technology. The results are summarized in Table 4. As shown in the table, the proposed C1-PET design achieves a maximum lift of 20.07 g, with a total system weight of 5.3 g, resulting in a lift-to-weight ratio of approximately 3.79, the performance is between that of RoboBee [20,21] and DelFly Nimble [22] products. Furthermore, the proposed flapping-wing mechanism operates at a moderate flapping frequency of 20.27 Hz, eliminating the need for high-frequency actuation. This not only helps to reduce mechanical wear but also simplifies control complexity.

Overall, the C1-PET configuration is competitive in terms of aerodynamic efficiency, structural simplicity and practicality, demonstrating the application potential of the flapping mechanism designed in this paper in the micro aircraft market.

## 5. Conclusions

In order to investigate the influence of T800 composite materials and different wing membrane materials on the mechanical properties of the flapping mechanism, this paper designs a single-degree-of-freedom flapping mechanism. Based on the different distribution patterns of T800 carbon fiber rods on the wing membrane surface, three wing structures are developed. Mechanical property comparison experiments are conducted by combining three different wing membrane materials, and the following conclusions can be drawn.

1. By introducing the T800 carbon fiber composite material-designed biomimetic “wing membrane-wing vein” structure in specific areas of the wing membrane, the local structural stiffness was effectively enhanced, and the structural vibration was suppressed, thereby improving the stability of flapping-wing flight.
2. Under low-voltage driving conditions, although the semi-covered structure has lower stiffness, its lift performance is superior to the other two structures, demonstrating good lightweighting and lift balance, and having certain potential for engineering applications.
3. Under the same configuration, the lift performance of the wing surface using PET membrane material is significantly better than that of polyimide membrane and kite fabric materials.
4. The mechanical property data of the three types of wing membrane materials (C2 and C3) indicate that in engineering practice, a solution can be adopted that combines the artificial wing membrane-wing vein structure with lightweight and high-performance wing membrane materials to enhance the aerodynamic performance and structural reliability of micro flapping-wing aircraft.

## Figures and Tables

**Table 1:** Properties of three types of wing membrane materials.

Material Type	Density (g/cm^3^)	Tensile Strength (MPa)	Elastic Modulus (GPa)	Elongation at Break (%)	Surface Energy (mJ/m^3^)
PET	1.38	50–80	2–4	50–150	40–45
polyimide	1.42	150–300	2.5–4	20–50	40–50
kite fabric	0.9–1.2	100–300	_	10–30	_

**Table 2:** Lift data from three-wing membrane three-configuration force measurement experiments.

Voltage	C1-PET	C2-PET	C3-PET	C1-PI	C2-PI	C3-PI	C1-Kite	C2-Kite	C3-Kite
1 V	5.07 g	1.01 g	1.09 g	2.91 g	1.05 g	0.66 g	3.02 g	1.19 g	1.38 g
2 V	11.47 g	5.66 g	5.05 g	7.36 g	5.69 g	3.11 g	6.87 g	5.36 g	3.92 g
3 V	16.48 g	11.69 g	7.24 g	12.25 g	8.25 g	6.09 g	11.32 g	9.32 g	6.66 g
4 V	20.07 g	17.40 g	11.25 g	14.04 g	12.87 g	9.18 g	14.77 g	10.39 g	9.01 g

**Table 3:** Frequency data from three-wing membrane three-configuration force measurement experiments.

Voltage	C1-PET	C2-PET	C3-PET	C1-PI	C2-PI	C3-PI	C1-Kite	C2-Kite	C3-Kite
1 V	8.94 Hz	4.75 Hz	6.44 Hz	10.07 Hz	4.56 Hz	5.21 Hz	7.97 Hz	5.21 Hz	4.88 Hz
2 V	13.98 Hz	8.59 Hz	11.32 Hz	14.69 Hz	8.53 Hz	10.41 Hz	10.93 Hz	9.17 Hz	8.98 Hz
3 V	17.76 Hz	12.43 Hz	13.01 Hz	17.56 Hz	12.37 Hz	11.95 Hz	13.90 Hz	13.15 Hz	12.11 Hz
4 V	20.27 Hz	15.88 Hz	15.77 Hz	18.66 Hz	15.95 Hz	14.28 Hz	16.87 Hz	16.17 Hz	14.84 Hz

**Table 4:** The performance comparison of the flapping mechanism designed in this paper with Robobee and Defly.

Voltage	Voltage (V)	Lift (g)	Weight (g)	Lift to Weight	Flapping Frequency (Hz)
C1-PET	4.3	20.07	5.3	3.79	20.27
Robobee	100	0.7	0.175	4	120
Defly Nimble	8.4	45	29	1.55	17

## Data Availability

The datasets presented in this article are not readily available because these data are part of the ongoing research. Requests to access the datasets should be directed to corresponding authors.
